# Histone deacetylase inhibition sensitizes osteosarcoma to heavy ion radiotherapy

**DOI:** 10.1186/s13014-015-0455-z

**Published:** 2015-07-16

**Authors:** Claudia Blattmann, Susanne Oertel, Markus Thiemann, Anne Dittmar, Eva Roth, Andreas E. Kulozik, Volker Ehemann, Wilko Weichert, Peter E. Huber, Albrecht Stenzinger, Jürgen Debus

**Affiliations:** Department of Radiation Oncology, University of Heidelberg, Heidelberg, Germany; Department of Pediatric Oncology, Hematology and Immunology, University Children’s, Hospital of Heidelberg, Heidelberg, Germany; Institute of Pathology, University of Heidelberg, Heidelberg, Germany; Department of Radiation Oncology, German Cancer Research Center, Heidelberg, Germany; German Cancer Consortium (DKTK), Heidelberg, Germany; National Center for Tumor Diseases (NCT), University of Heidelberg, Heidelberg, Germany; Pädiatrie 5, Olgahospital, Kriegsbergstr. 62, 70174 Stuttgart, Germany

**Keywords:** Osteosarcoma, Carbon ion radiotherapy, Histone deacetylase inhibition, Suberoylanilide hydroxamic acid, Radiosensitization, Mouse model

## Abstract

**Background:**

Minimal improvements in treatment or survival of patients with osteosarcoma have been achieved during the last three decades. Especially in the case of incomplete tumor resection, prognosis remains poor. Heavy ion radiotherapy (HIT) and modern anticancer drugs like histone deacetylase inhibitors (HDACi) have shown promising effects in osteosarcoma *in vitro*. In this study, we tested the effect of HIT and the combination of HIT and the HDACi suberoylanilide hydroxamic acid (SAHA) in a xenograft mouse model.

**Methods:**

Osteosarcoma xenografts were established by subcutaneous injection of KHOS-24OS cells and treated with either vehicle (DMSO), SAHA, HIT or HIT and SAHA. Tumor growth was determined and tumor necrosis, proliferation rate, apoptotic rate as well as vessel density were evaluated.

**Results:**

Here, we show that the combination of HIT and SAHA induced a significant delay of tumor growth through increased rate of apoptosis, increased expression of p53 and p21^Waf1/Cip1^, inhibition of proliferation and angiogenesis compared to tumors treated with HIT only.

**Conclusion:**

HIT and in particular the combination of HIT and histone deacetylase inhibition is a promising treatment strategy in OS and may be tested in clinical trials.

## Background

Osteosarcoma (OS) is the most frequent primary malignant bone tumor in children and adolescents. They account for up to 15 % of all extracranial solid neoplasms in patients aged 15–19 years. Despite significant advancements in the diagnosis and treatment to date, overall survival has remained relatively constant for about three decades and is still poor for non-resectable tumors and advanced metastatic disease [1]. Until now the only therapy that is validated in a large number of patients is complete tumor resection combined with neo-adjuvant and adjuvant chemotherapy to establish local control. With this therapy regimen, the survival rate for patients with localized osteosarcoma is in the range of 60–70 %, for patients with metastases in the range of 15–30 %. However, there is also a considerable fraction of patients (about 10 %) with non-resectable tumors. Moreover surgery is often denied by patients when a complete surgical resection can only be reached by amputation of a limb. In these cases radiation therapy and chemotherapy is a possible option that has been validated in a limited number of patients [2]. Radiotherapy in this setting is still being discussed controversially: particularly for children since high radiation doses are required, bearing unwanted side-effects including acute and late local toxicity and the considerable risk for secondary malignancies. Therefore novel therapeutic approaches like irradiation with heavy ions (HIT) or the use of radiation sensitizers that selectively augment the response of osteosarcomas to radiation and thus increase the therapeutic ratio are urgently needed.

HIT offers significant advantages in comparison to conventional photon therapy (XRT). The physical depth-dose distribution in tissue of heavy ions is characterized by a small entrance dose and a distinct maximum (Bragg peak) near the end of range with a sharp fall-off at the distal edge. These facts allow delivery of the dose with millimeter precision. In addition, HIT exhibit an enhanced biological effectiveness in the Bragg peak region caused by the dense ionization of individual particle tracks resulting in reduced cellular repair. These advantages will potentially reduce undesirable side-effects and the risk of secondary malignancies as well as improve outcome and quality of life. Therefore, it makes them particularly attractive for the treatment of pediatric patients and young adults as well as for radio-resistant tumors such as osteosarcomas [3–6].

Histone deacetylase inhibitiors (HDACi) are epigenetic modulators with manifold effects on tumor cells [7]. In a number of *in vitro* and *in vivo* experimental models, HDACi have been shown to act as radiosensitizers in a number of solide tumors including gliomas, colorectal carcinomas and melanoma [8–11]. The HDACi suberoylanilide hydroxamic acid (SAHA) has been approved for the treatment of T-cell lymphoma. Furthermore, promising effects of SAHA in solide tumors have been shown clinical trials [12]. Recently, we showed a significant radiosensitizing effect of SAHA in combination with XRT in osteosarcoma *in vivo* [13]. First *in vitro* data showed promising effects by the combination HIT and SAHA in infantile sarcoma cell lines [14,15].

The aim of the present study was to evaluate the efficacy of HIT alone and in combination with SAHA as well as potential underlying mechanisms in osteosarcomas *in vivo*.

## Methods

### Cells and reagents

The KHOS-24OS human OS cell line was purchased from the American Type Culture Collection (ATCC; Rockville, MD) and maintained in complete culture medium (DMEM) supplemented with 10 % FCS. SAHA was obtained from Alexis Biochemicals (Lörrach, Germany), DMSO from Carl Roth Biochemicals (Karlsruhe, Germany). Ketamine (0.4 mg/20 g BW) and xylazine (Bayer Germany) (90 mg/20 g BW) were used to anesthetize mice during radiation treatments.

### Animal model

All animal experiments were approved by the ethics committee of the “Regierungspräsidium Karlsruhe” (reference number 35-9185.81/G-165/08).

For the pilot experiment were 30 mice used to define the HIT dose: control group and irradiated mice with 2 Gy, each with 5 mice, irradiated mice with 1, 3, 5, 10 and 20 Gy, each with 4 mice.

The number of mice for the main experiment was calculated with 24 mice per treatment group. Xenografts of human KHOS-24OS cells were established by subcutaneous inoculation of tumor fragments (diameter 1–2 mm) into the hind legs of 10 weeks old SCID mice (CB17/Icr-*Prkdc*^*scid*^/IcrCrl) (Charles River, Wilmington, Mass.). The tumor fragments were generated from a donor animal after inoculation of 5 x 10^6^ KHOS-24OS cells into the hind legs. When the tumor reached a size of about 14 ×e 14 mm, they were fragmented and implanted into all experimental animals. In a preliminary dose finding trial 6 different doses between 1 and 20 Gy of carbon ions were given and compared to a control group. Every group consisted of 5 mice. The mice were maintained under specific pathogen-free conditions, food and water were supplied ad libidum. Housing and all procedures involving the mice were performed according to the protocols approved by the local regional board.

When tumors reached 90–100 mm^3^, mice were randomly assigned to one of four treatment groups: vehicle (DMSO) control (24 animals), SAHA alone (21 animals), radiation alone (20 animals), or combined SAHA and radiation (21 animals). In total, 10 of 96 animals died during the investigation period due to anesthesia-related complications (3 mice in the SAHA group, 4 mice in the XRT group and 3 mice in the combination treatment group. Therefore, these groups are smaller than the control group. SAHA 100 mg/kg was solubilized in DMSO (99.5 %), given intraperitoneally (i.p.), started 24 h before radiation and applied for 5 days a week, 3 weeks in total. On day 1, mice tumors were irradiated with carbon ions in a single dose of 2.5 Gy. For HIT, mice were anesthetized with ketamine and xylazine and then exposed to HIT directed at the tumor site. In the following, the mice were weighed, and the tumor sizes were measured using a caliper twice a week. Tumor length (L) and width (W) were measured and tumor volume calculated as (L × W^2^/2), where L = longest diameter and W = shortest diameter. For each tumor, tumor size and multiplication to initial tumor size was assessed and documented. Animals were euthanized by cervical dislocation when tumors reached a volume of 2.0 cm^3^ or severe necrosis latest 45 days after treatment start.

### Immunohistochemical analysis

Immunohistochemistry was performed in three tumors per treatment group at the following time points: 24 h, 8 days, 24 and 45 days after HIT.

3 micrometre thick whole tumor sections were cut from formalin-fixed, paraffin-embedded (FFPE) tissue blocks. Sections were stained with hematoxylin-eosin (HE; Sigma-Aldrich, St Louis, USA). On these H&E stained slides, the total amount of necrotic versus vital tissue was analyzed in 5 % increments and means for all treatment groups were calculated.

Immunohistochemical staining for the evaluation of proliferative capacity and microvessel density was carried out with anti- Ki-67 (monoclonal mouse clone MIB-1, 1:200; Dako, Hamburg, Germany) and anti- CD34 (1:25, pH6; Dako, Hamburg, Germany) *antibodies* as recommended by the manufacturer.

Ki-67-positive tumor cell nuclei were counted in 5 representative areas of each tumor. Evaluation of vessel density was done on the slides stained for CD34. The two most vascularized areas within a given tumor (‘hot spots’) were chosen at low magnification (×10) and vessels were counted in a representative high magnification (× 400; image size 385.9 × 251 μm) field in each of these two areas. Single immunoreactive endothelial cells, or endothelial cell clusters separate from other microvessels, were counted as individual microvessels. Endothelial staining in large vessels with tunica media, and nonspecific staining of nonendothelial structures, was disregarded.

Apoptosis was detected by an *in situ* apoptosis detection kit, ApopTAG^®^ (S7100; Chemicon International (Millipore), Temecula, CA, USA) on 3 μm thick formalin-fixed, paraffin-embedded whole tumor sections as specified by the manufacturer. TUNEL-positive cells were counted at 40x magnification 5 representative areas of each tumor.

For protein detection, the following primary antibodies were employed: rabbit monoclonal anti-p21 (1:100, pH9, Abcam, Cambridge, UK; #ab92675) and anti-p53 (1:100, pH9, Dako, Hamburg, Germany. After heat-induced antigen retrieval, slides were incubated with primary antibody at 4 °C overnight. Bound antibody was detected by a Super Sensitive IHC-Detection- System (BioGenex, San Ramon, CA). For colour development, a DAB system (DAKO, Hamburg, Germany) was used. The scores of p21^Waf1/Cip1^ and p53 expression were calculated by multiplication of staining intensity and staining percentage. Staining intensity was defined as follows: 0 = no staining, 1 = weak staining, 2 = moderate and 3 = strong staining. Staining percentage was defined as follows: 0 = no cells, 1 = 1–10 % cells, 2 = 11–50 % cells, 3 = 51–80 % and 4 > 80 % cells. Evaluation of all tissue based parameters was done by two experienced pathologist (WW and AS) together on a multiheaded microscope. Both observers were blinded to the mouse treatment allocation.

### Data analysis

The two-sided *t*-test was used to analyze the differences between the treatment groups. *P* values < 0.05 were considered statistically significant. Data are presented either as mean or as median, error estimation was performed by calculation of the standard error of mean (SEM).

Actual tumor growth delay was calculated with (T’x-Tx)/Tx as the time it took the irradiated tumors (T’) and the tumors of the control group to x-fold multiply their volume. Local control was estimated by Kaplan-Meier curves, and the differences in time-to-failure (TTF) between groups were assessed using the log-rank test.

### Ethical statement

All authors confirm that the animal experiments comply with the “Animal Research: Reporting *In Vivo* Experiments” (ARRIVE) guidelines.

## Results

Previously, we assessed the potency of XRT and SAHA in osteosarcoma xenografts [13]. Now, HIT and SAHA were investigated using the same mouse model and results were compared.

### HIT is superior to XRT in OS xenografts

**Fig. 1:**
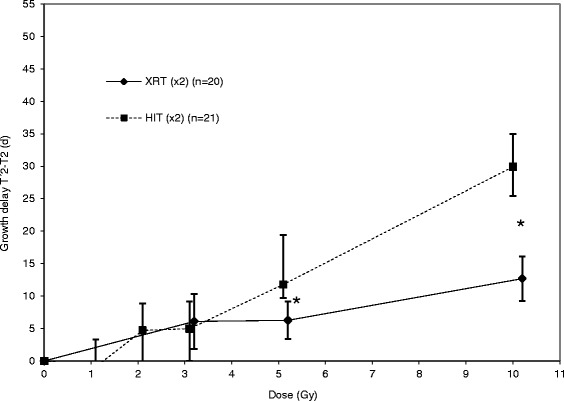
HIT is superior to XRT in osteosarcoma xenografts. Tumor duplication after treatment with different doses of HIT and XRT was compared in to animal groups. Actual tumor growth delay was calculated with (T’x-Tx)/Tx as the time taken for the irradiated tumors (T’) and the control tumors (T) to x-fold multiply their volume (x)

**Fig. 2:**
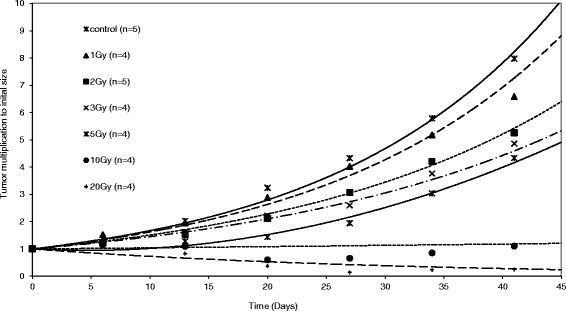
The HIT dosage of 2.5 Gy leads to a tumor growth delay of 14 days. In a preliminary test, xenografts were irradiated with a single dose of 1, 2, 3, 5, 10 or 20 Gy. The dose of 2.5 Gy were chosen for our further experiments because significant but not to effective growth retardation within the first 14 days after treatment was seen leaving capacity for the anticipated additional effect of SAHA

Our dosimetry trial revealed that a higher biologic effectiveness of HIT compared to XRT in our osteosarcoma model can be observed especially at higher single dose levels above 5 Gy (Fig. 1). On the basis of these data, for the combination trial a single dose of 2.5 Gy was picked in order to prevent from covering up a possible supraadditive effect. This dose showed significant growth retardation within the first 14 days after treatment, comparable to that observed after 5 Gy with XRT, but was not too effective to mask the anticipated additional effect of SAHA (Fig. 2).

### SAHA sensitizes OS to HIT *in vivo*

Mice bearing KHOS-24OS xenografts were injected i.p. with SAHA (100 mg/kg) for 5 days a week, 15 days in total, starting 24 h before HIT with a dosage of 2.5Gy.

SAHA given as a single-agent for 15 days induced no significant tumor growth delay compared to mice of the control group.

**Fig. 3:**
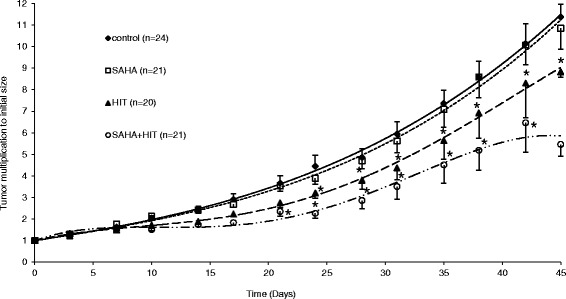
The combination of HIT and SAHA results in a significant tumor growth delay compared to treatment with HIT or SAHA only. Osteosarcoma xenografts were treated with DMSO (controls), suberoylanilide hydroxamic acid (SAHA), irradiation (HIT) or SAHA plus HIT and tumor growth was determined until day 45 after HIT. Comparing HIT as mono-treatment to SAHA only, HIT seemed to be superior from day 10 on after treatment start reaching significance at day 23. The combination of HIT and SAHA yielded a significant (*) tumor growth retardation compared to SAHA only and HIT only starting day 20 and day 25 respectively

In analogy to our recent publication about the combination of XRT with SAHA [16], the combination of HIT with SAHA led to a significantly delayed tumor growth rate compared to HIT alone (p_d23_ = 0.005). Comparing HIT as mono-treatment to SAHA only, HIT seemed to be superior from day 10 on after treatment start reaching significance at day 24 (p_d24_ = 0.04). The combination of HIT and SAHA yielded a significant tumor growth retardation (measured by tumor volume) compared to SAHA only and HIT only starting day 20 (p_d20_ = 0.005) and day 25 (p_d25_ = 0.04) respectively (Fig. 3).

**Fig. 4:**
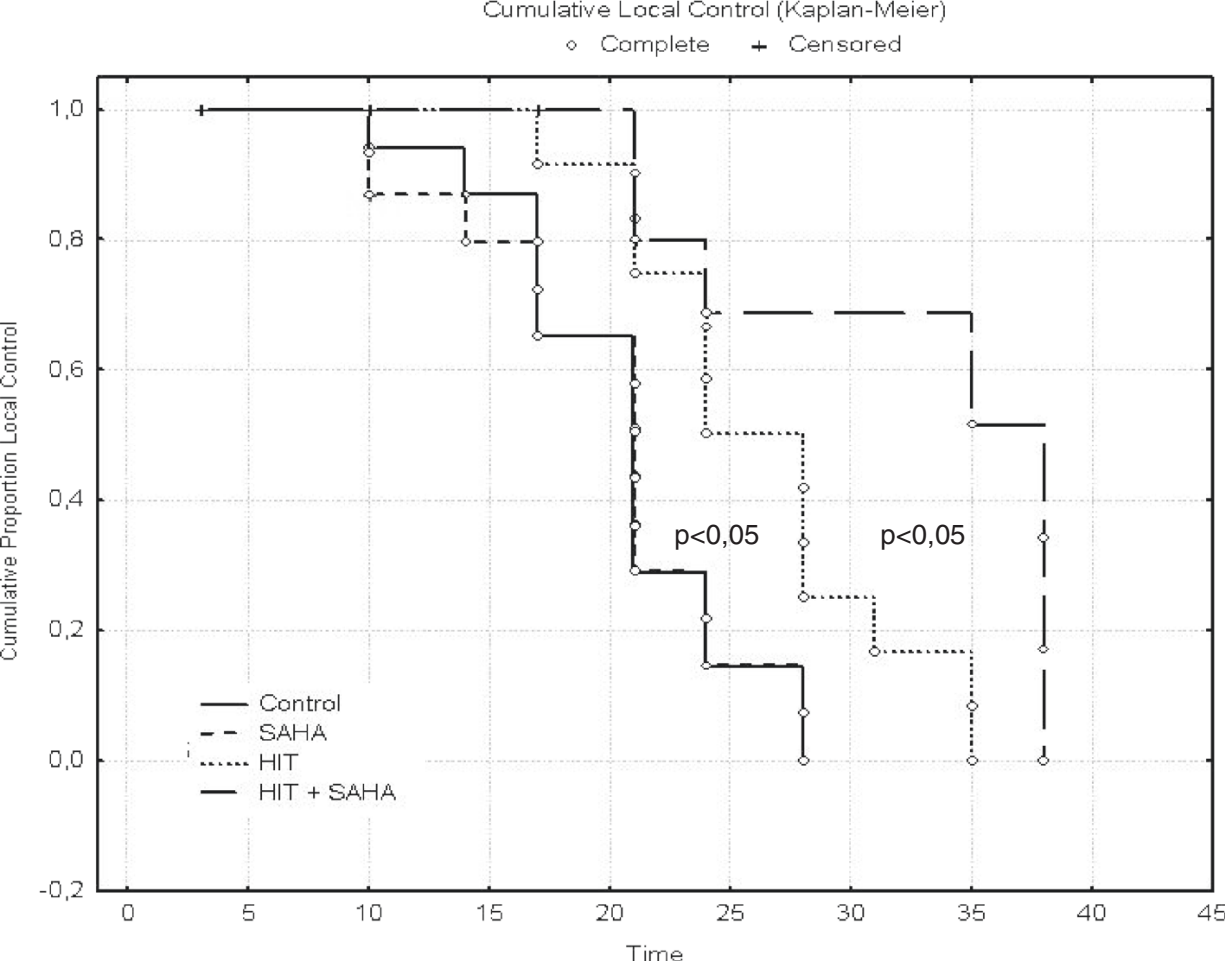
Combination of HIT and SAHA induces an increased local control. Tumor growth of osteosarcoma xenografts was determined after treatment with DMSO (controls), suberoylanilide hydroxamic acid (SAHA), irradiation (HIT) or SAHA plus HIT. Local control was defined as tumor growth > 1000 m^3^ and calculated according to the method of Kaplan and Meier. The combination of HIT and SAHA led to a significant local control compared to SAHA only and HIT only starting day

The significant (*p* < 0,05) superior effect of HIT and SAHA was most pronounced between days 25 and 35 after treatment as visualized after calculation according to Kaplan-Meier. As planned, neither treatment was dosed intensively enough to yield tumor control beyond 40 days (Fig. 4).

Heavy ion radiation and SAHA application were tolerated without observable toxicity in mice. In total, 4 of 86 animals died during the investigation period due to anesthesia-related complications. After treatment all mice were weighed once a week. Neither variation in mean weight between the four groups nor severe weight loss in any mice were observable.

Dose enhancement factor at triplication of tumor volume was 1.7 in the SAHA plus HIT treated group, 1.4 in the HIT treated group and 1.1 and in the SAHA treated group.

### The combination of HIT and SAHA inhibits proliferation

Analysis of tumors was performed 24 h, as well as 8, 24 and 45 days after HIT.

**Fig. 5:**
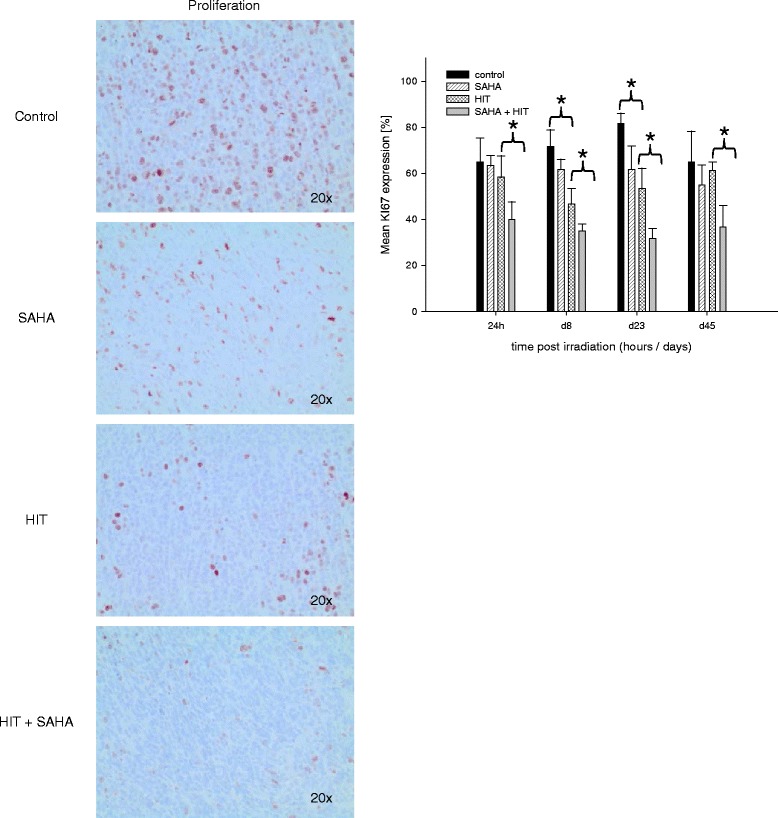
Treatment with HIT and SAHA results in a reduced proliferation in osteosarcoma xenografts. Histological analysis of proliferation in tumors treated with DMSO (control), suberoylanilide hydroxamic acid (SAHA), irradiation (HIT) or HIT and SAHA. The proliferation rate was significantly reduced in tumors after treatment with SAHA and HIT at all investigation time points. HIT only treatment led to a significantly lower proliferation rate 8 and 24 days after irradiation compared to the control group. SAHA only treatment had no significant effect on tumor proliferation

The proliferation rate was significantly reduced in tumors after treatment with SAHA and HIT at all investigation time points. HIT only treatment led to a significantly lower proliferation rate 8 and 24 days after irradiation compared to the control group. SAHA only treatment had no significant effect on tumor proliferation (Fig. 5).

### HIT and SAHA treatment induces apoptosis and necrosis

**Fig. 6:**
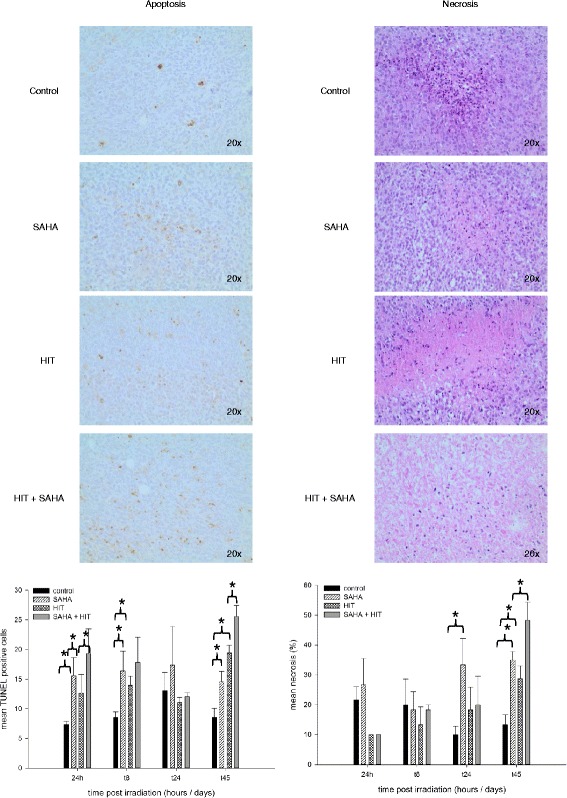
Impairment of apoptosis and necrosis after HIT and SAHA treatment. Apoptosis and necrosis in osteosarcoma xenografts were analyzed after treatment with DMSO (control), suberoylanilide hydroxamic acid (SAHA), irradiation (HIT) or HIT and SAHA. The combination treatment lead to a significant (*) induction of apoptosis one day, 8 and 45 days after HIT compared to HIT only treatment. Apoptosis was also increased after SAHA only and HIT only 24 h, 8 and 45 days after irradiation but not as much as treatment with HIT and SAHA. Combination of HIT and SAHA lead to a significant (*) induction of necrosis on day 45. SAHA only and HIT only treatment resulted in a significantly higher rate of necrosis from day 24 on but not at earlier time points compared to the control groups

The TUNEL assay showed a significant induction of apoptosis after treatment with HIT and SAHA compared to HIT only treatment 1, 8 and 45 days (*p* = 0.002) after HIT. Apoptosis was also increased after SAHA only and HIT only 24 h, 8 and 45 days after irradiation but not as much as treatment with HIT and SAHA (Fig. 6).

**Fig. 7:**
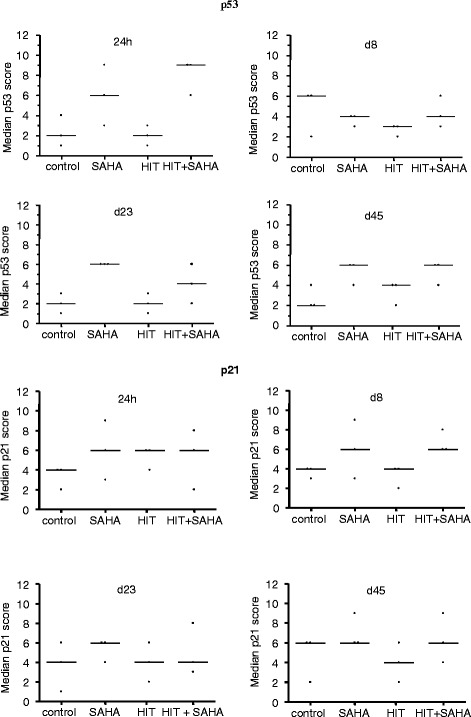
Expression of p53 and p21^WAF1/CIP1^ is impaired in osteosarcoma xenografts after treatment with HIT and SAHA. Expression of p53 and p21^WAF1/CIP1^ was analyzed after treatment with DMSO (control), suberoylanilide hydroxamic acid (SAHA), irradiation (HIT) or HIT and SAHA. p53 expression was increased in all SAHA treated tumors compared to the vehicle treated control and only irradiated tumors on day 23 and 45. Increased p21^Waf1/Cip1^ expression was detected in tumors after SAHA only and SAHA and HIT treatment compared to the controls and the HIT treated tumors on day 8

We further analyzed expression of p53 and p21^Waf1/Cip1^ in three tumors per treatment group. Our results revealed a higher p53 expression in all SAHA treated tumors compared to the vehicle treated control and only irradiated tumors on day 24 and 45. p21^Waf1/Cip1^ expression was increased in tumors after SAHA only and SAHA and HIT treatment compared to the controls and the HIT treated tumors on day 8 but not at other points of time (Fig. 7).

After treatment with SAHA only, the rate of necrosis was significantly increased on day 45 compared to the controls but not before (Fig. 7).

Combination of HIT and SAHA lead to a significant induction of necrosis on day 45 (*p* = 0.01). Furthermore, SAHA only and HIT only treatment resulted in a significantly higher rate of necrosis from day 24 on but not at earlier time points compared to the control groups (Fig. 6). However, rate of necrosis was highest in the combination treatment group.

### SAHA significantly impairs angiogenesis in combination with HIT

**Fig. 8:**
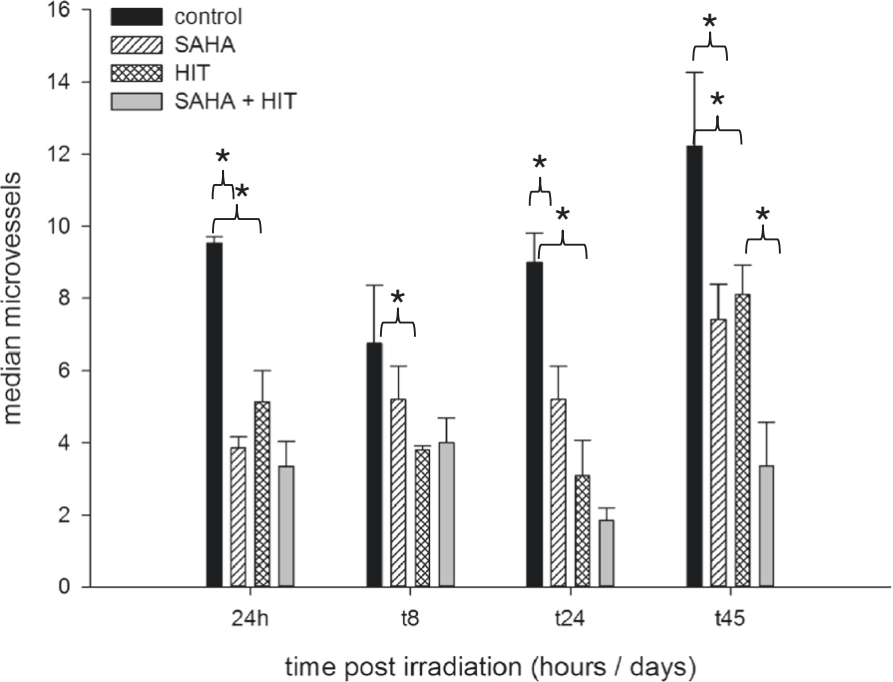
Density of microvessels is significantly reduced in osteosarcoma after combination treatment. Xenografts were treated with DMSO (control), suberoylanilide hydroxamic acid (SAHA), irradiation (HIT) or HIT and SAHA. The density of microvessels was reduced in tumors of all treatment groups at all investigation time points. Tumors treated with HIT and SAHA showed a significant (*) lower vascularization compared to tumors treated with HIT from day 24 on and the lowest vascularization at all

We also evaluated the number of microvessels by CD34 immunohistochemistry. Compared to the control group, the density of microvessels was reduced in tumors of all treatment groups at all investigation time points. Tumors treated with HIT and SAHA showed a significant lower vascularization compared to tumors treated with HIT from day 24 on (*p* = 0.02) and the lowest vascularization at all (Fig. 8).

## Discussion

Osteosarcoma are highly malignant bone tumors and rather resistant to radiotherapy. Prognosis is fatal whenever local control cannot be achieved by surgery or/and radiotherapy [17,18]. Therefore, for patients with inoperable or not completely resectable osteosarcoma new treatment strategies with novel irradiation techniques like proton or heavy ion beams or new therapeutic substances with radiosensitizing effects are under investigation. HDACi have been shown to be effective anticancer agents [12]. In solid tumors, the full therapeutic potential of HDACi can, however, only be achieved in combination with other agents and / or radiation [19,20]. SAHA is one of the well-established HDACi and has been shown to act as a radiosensitizer *in vitro* and *in vivo* in different tumor entities [7–10,12,13]. The current study is the first that investigates the efficacy of HDACi in combination with HIT, the most effective form of radiation in osteosarcoma in clinical practice [21].

Comparing the current study with the results of our recently published study [12], we observed a higher efficacy of HIT at higher single doses > 5 Gy. Although these data correspond well to previous findings, this observation needs to be reevaluated as the rate of growth in the control animals of the two subsequently performed studies varied considerably.

Furthermore, our study clearly shows that SAHA significantly and effectively radiosensitizes osteosarcoma to HIT *in vivo* and that the combination of SAHA and HIT is superior to either agent alone. Growth of tumors treated with SAHA and HIT was significantly delayed compared to tumors treated with either HIT only or SAHA without observable toxicity in our mice.

Immunhistochemical analysis of tumor xenografts revealed a significant increase of necrosis on day 45 after HIT and SAHA treatment compared to HIT alone. Proliferation was impaired in tumors after combination treatment compared to tumors treated with HIT only. Furthermore, apoptosis was significantly induced by treatment with HIT and SAHA compared to irradiated tumors.

Our results are in line with the observations of many other studies [22–25]. HDACi are regarded as agents capable of reactivating apoptosis in tumor cells by affecting very different parts of the apoptosis cascade; for example, through increased expression of death receptors like tumor necrosis factor-related apoptosis-inducing ligand (TRAIL) cell surface receptors or by directly stimulating a death receptor pathway without altered receptor expression [26]. Henderson et al. reported about HDACi - induced cell death by activation of caspases but also could show that inhibition of caspases did not block HDACi - induced cell death [27]. Furthermore, apoptosis-inducing effects of HDACi might not only be due to histone deacetylation and subsequent transcriptional regulation. HDACi can also block deacetylation of important proteins, such as the tumor suppressor gene TP53 [28]. P53 is one of the most commonly altered transcription factors in cancer and plays a pivotal role in the cellular response to DNA-damaging agents [29]. A number of studies demonstrate activation of p53 in cells after exposure to HDACi and ability of HDACi to enhance radiation response in cancer cells through increase of p53 acetylation-phosphorylation [30–32]. Correspondingly, we were able to observe an increase of p53 expression in all SAHA treated samples. However, there was no difference between SAHA only and SAHA plus HIT treated tumors and no supraadditive effect that might prove a sensitization to HIT beyond the SAHA only effect.

HDACi-mediated effects on the cell cycle are also postulated to be a key reason for their toxicity in tumor cells. Most HDACi lead to a cell cycle arrest at G1 associated with induction of CDKN1A/p21^WAF1/CIP1^ [33]. Recently, we observed a HDACi-induced cell cycle arrest at G1 accompanied with up-regulation of p21^WAF1/CIP1^*in vitro*. However, this effect does not fully explain the radiosensitizing property because multiple underlying mechanisms are described like effects on DNA damage repair, angiogenesis and apoptosis.

Solid tumors are frequently angiogenesis dependent. The immunohistochemical assessment of angiogenesis in our animal model showed a significant decrease of microvessels in all tumors treated with HIT and SAHA compared to tumors treated with either HIT or SAHA. Vascularization of malignant lesions depends on the expression of specific genes in both endothelial and tumor cells. There is evidence that several members of the histone deacetylase family play key roles in the regulation of these genes. Indeed, numerous *in vitro* and *in vivo* studies demonstrated that inhibitors of HDAC modulate angiogenic gene expression in both endothelial and cancer cells and disturb the delicate and complex balance between the collective action of pro-angiogenic factors and angiogenesis inhibitors [34].

## Conclusions

Taken together, we demonstrate that SAHA leads to a significant tumor growth delay of OS *in vivo* when combined with HIT. This effect in our *in vivo* model is most likely caused by a combination of an induction of apoptosis, an impairment of angiogenesis and a supraadditive impact of the combination treatment on proliferation. Our results indicate that a combination of HDACi and HIT might be a strategy therapeutic option for patients with OS, particularly if tumors are non-resectable.

## Abbreviations

HDACi: Histone deacetylase inhibitor
HIT: Heavy ions
SAHA: Suberoylanilide hydroxamic acid
XRT: Conventional photon therapy
